# The association between socioeconomic status and disability after stroke: Findings from the Adherence eValuation After Ischemic stroke Longitudinal (AVAIL) registry

**DOI:** 10.1186/1471-2458-14-281

**Published:** 2014-03-26

**Authors:** Janet Prvu Bettger, Xin Zhao, Cheryl Bushnell, Louise Zimmer, Wenqin Pan, Linda S Williams, Eric D Peterson

**Affiliations:** 1 Duke School of Nursing and Duke Clinical Research Institute, 307 Trent Drive, 511 DUMC 3322, Durham, NC 27710, USA; 2 Duke Clinical Research Institute, Durham, NC 27710, USA; 3 Wake Forest Baptist Medical Center, Winston-Salem, NC, USA; 4 Roudebush Veterans Affairs Medical Center, Indianapolis, IN, USA

**Keywords:** Ischemic stroke, Recovery, Disability, Socioeconomic position, Outcomes research

## Abstract

**Background:**

Stroke is the leading cause of disability among adults in the United States. The association of patients’ pre-event socioeconomic status (SES) with post-stroke disability is not well understood. We examined the association of three indicators of SES—educational attainment, working status, and perceived adequacy of household income—with disability 3-months following an acute ischemic stroke.

**Methods:**

We conducted retrospective analyses of a prospective cohort of 1965 ischemic stroke patients who survived to 3 months in the Adherence eValuation After Ischemic stroke – Longitudinal (AVAIL) study. Multivariable logistic regression was used to examine the relationship of level of education, pre-stroke work status, and perceived adequacy of household income with disability (defined as a modified Rankin Scale of 3–5 indicating activities of daily living limitations or constant care required).

**Results:**

Overall, 58% of AVAIL stroke patients had a high school or less education, 61% were not working, and 27% perceived their household income as inadequate prior to their stroke. Thirty five percent of patients were disabled at 3-months. After adjusting for demographic and clinical factors, stroke survivors who were unemployed or homemakers, disabled and not-working, retired, less educated, or reported to have inadequate income prior to their stroke had a significantly higher odds of post-stroke disability.

**Conclusions:**

In this cohort of stroke survivors, socioeconomic status was associated with disability following acute ischemic stroke. The results may have implications for public health and health service interventions targeting stroke survivors at risk of poor outcomes.

## Background

Every 40 seconds someone in the United States (U.S.) experiences a stroke [1]. The total cost of care, medications and missed days of work were estimated at $73.7 billion for the U.S. in 2010; the costs to stroke survivors and their families are immeasurable. Stroke is the leading cause of long-term disability among adults. Residual impairment and disability are present in up to 75% of stroke survivors and 15-30% of stroke survivors report a significant level of disability [1]. Stroke patients requiring constant care three months following their stroke have nearly a seven-fold increased risk in mortality twelve months post-stroke [2]. Identifying patients at greater risk for poor three month outcomes has significant implications for services and systems aimed to support recovery in the post-acute period.

Socioeconomic status (SES) can affect survival and disease prognosis for many conditions. The relationship between higher social class and improved functional status after myocardial infarction, for example, was reported more than 15 years ago [3]. To date, however, there have been few studies that have examined whether functional outcomes including disability following stroke are affected by patients’ SES [4]. Previous investigations had limited measures of SES, examined aggregate rather than patient level SES [4], and studied non-U.S. populations.

The purpose of this study was to examine the relationship of three indicators of SES—educational attainment, pre-stroke working status and perceived adequacy of household income—with disability 3-months following an acute ischemic stroke. We also sought to determine the relationship of SES and post-stroke disability when all three indicators were examined collectively, adjusting for demographic and clinical factors. Our study hypothesis was that stroke survivors who were not working pre-stroke, reported inadequate income pre-stroke, and had less education would require assistance with basic or instrumental activities of daily living as measured with the modified Rankin Scale (mRS) at 3-months.

## Methods

The Adherence eValuation After Ischemic stroke-Longitudinal (AVAIL) study was a prospective national cohort study implemented in collaboration with the American Heart Association’s Get With The Guidelines-Stroke (GWTG-Stroke) program. GWTG-Stroke is an ongoing, voluntary, registry and performance improvement initiative for acute hospitals that collect patient level data on characteristics, diagnostic testing, treatments, adherence to evidence-based standards of care, and in-hospital outcomes in patients hospitalized with stroke [5]. Outcome Sciences, Inc. serves as the data collection coordinating center for GWTG. The Duke Clinical Research Institute served as the data collection coordinating center for AVAIL and serves as the data analysis center for both GWTG-Stroke and AVAIL.

AVAIL was designed to examine medication adherence, clinical, and functional outcomes at 3 and 12 months after an adult had a transient ischemic attack or ischemic stroke [6]. All participating hospitals were required to obtain local institutional review board approval before study initiation in addition to institutional review board approval obtained at the study coordinating and data analysis center at Duke University.

### Population

Of the 676 hospitals participating in GWTG-Stroke at the time of recruitment (2006), 109 initially agreed to participate and 106 hospitals ultimately enrolled patients. Study personnel screened patients for AVAIL eligibility during the acute hospitalization and then obtained informed consent. To be eligible, adults age 18 years and older had to be hospitalized at a GWTG-Stroke participating hospital with a primary diagnosis of transient ischemic attack or ischemic stroke based on International Classification of Diseases Ninth Edition codes (435.x or 433.x, 434.x and 436, respectively), able to provide informed consent or have a legally authorized representative sign, and have patient data documented in the GWTG-Stroke registry. AVAIL excluded patients with a diagnosis of subarachnoid or intracerebral hemorrhage or who were expected to survive six months or less. AVAIL participants with a transient ischemic attack (n = 570) were ineligible for this study of disability among ischemic stroke survivors and were additionally excluded. Compared to patients in the GWTG-Stroke participating hospitals, those in the AVAIL cohort were younger (AVAIL mean age 65.5 years vs. GWTG-Stroke 70.0 years), with a higher proportion male (55.7% vs. 46.6%), white (82.8% vs. 73.1%), and less severe stroke (National Institutes of Health Stroke Scale or NIHSS interquartile range 1–7 vs. 1–10).

### Data collection

Details of the design and conduct of GWTG-Stroke have been previously described [7,8]. At enrollment for AVAIL consented patients provided contact information for themselves, alternate contacts, and their primary physician. Among the baseline data additionally collected by sites for AVAIL [6], we included in this study the specific measures of SES (education, income and employment) that are not available in GWTG-Stroke.

#### *Indicators of socioeconomic status*

Level of educational attainment was reported as the highest grade or number of years completed in school. Patients who completed a high school degree or less were considered to have low educational SES. Work status prior to stroke was divided into four categories based on financially comparable households and social connectedness: working (full time, part time, or temporarily on sick leave); retired; disabled and not-working; and homemaker or unemployed (unemployed or looking for work). Patients who were retired, disabled and not-working, or unemployed/homemakers were considered to have lower SES by employment than patients who were employed and working prior to the acute stroke hospitalization. Since income information is considered to be sensitive, at risk for social desirability bias [9], and not a proxy for wealth [10], patients in AVAIL were asked to consider their household income from all sources prior to their hospitalization and report how well it met their basic needs on a four point scale [11]. Patients who reported that their income only somewhat or not at all met their basic needs were considered to have lower SES.

#### *Outcome measure: disability*

Patients were contacted by telephone 3 months after discharge from their acute hospitalization. The patient enrolled was the targeted respondent but when it was not possible to speak to the patient, when the information provided by the patient was deemed unreliable by the interviewer, or when the patient could not verbally respond, proxy interviews were conducted. Trained research personnel used standard scripts to conduct the telephone interview that included primarily standardized assessments [6]. The mRS was used in this study as a global measure of disability that provides six distinct stages of functioning [12]. Patients or their proxies were asked to respond yes or no for whether assistance was needed for each mRS level (0 = no assistance needed; 5 = required constant care) and the corresponding score was recorded. A mRS score of 3–5 (assistance needed with instrumental or basic activities of daily living, or constant care was required) was defined as post-stroke disability, the primary outcome of interest. We explored death and dependence (mRS score of 3–6) as a secondary outcome to include those patients who died prior to the 3-month AVAIL interview (N = 57).

### Analyses

Patient characteristics and measures of SES were compared between stroke patients with a mRS score of 0–2 and 3–5. P-values are based on Pearson chi-square tests for all categorical variables and chi-square rank based group means score statistics (equivalent to Wilcoxon tests) for all continuous/ordinal variables. All tests were 2-sided and calculated by comparing only non-missing values.

To evaluate the association between SES and disability (mRS score 3–5), a five-step strategy was used for the logistic regression models by adding covariates step by step. We first fit three unadjusted models, one for each measure of SES (education, work status and perceived income), using binomial logistic regression (step 1). We then sequentially added variables to each of the models. We adjusted each of the SES models for individual demographic characteristics of age, gender, and race (step 2). We then adjusted each of the SES models for demographic and clinical characteristics: medical history of prior stroke or transient ischemic attack, diabetes mellitus, hypertension, and smoking, treatment with tPA, stroke severity (NIHSS), and ability to ambulate at discharge (step 3). The step 3 models were restricted to those patients with a documented NIHSS score (N = 1380). Disability at 3-months was not different for patients with and without a documented NIHSS score. The three models in step 3 provided the independent association of each measure of SES with disability after adjusting for demographic and clinical characteristics. The next model included the three indicators of SES (step 4). We then examined the interaction of each indicator of SES with age and gender and found the interaction between income and age was significant in the model (p = 0.002). The final model examined all three SES measures adjusting for demographic and clinical characteristics and the interaction of income and age (step 5).

The examination of patient characteristics and the association of SES with the outcome were repeated for our secondary outcome of death and dependence (mRS = 3-6) to include the 57 patients who had died prior to the 3-month AVAIL interview (coded as a mRS = 6) and had data available for the independent variables.

Restricted cubic splines were used to examine linearity between continuous variables (age and NIHSS) and the outcome. Consequently, age when included in a model was examined as a 3-level categorical variable. Collinearity was examined in the final model using the variance inflation factor (VIF). Statistical analyses were performed using SAS 9.2, using the LOGISTIC procedure.

## Results

Of the 2,240 ischemic stroke patients alive 3-months after hospital discharge we excluded those patients missing mRS at 3-months (n = 117 follow-up not done and n = 31 missing mRS at 3-months) or SES information at baseline (n = 127). The demographic and clinical characteristics of the patients excluded were not different from those in our final study sample with the exception of those excluded being younger in age (median age 62 years vs. 66 years for study population, p < 0.001). Our final sample had 1,965 ischemic stroke patients from 98 hospitals. These patients were predominantly white, male, with a median age of 66 years (IQR 56–76 years) and an initial NIHSS score of 5 (IQR 1–7). The most common comorbidities were history of hypertension, diabetes mellitus, and prior stroke or transient ischemic attack. At discharge, 61.5% were ambulating independently and 59.6% were discharged home (Table 1).

**Table 1:** Patient characteristics associated with functional status at 3-months (N, %)

**Variable**	**Total N = 1965**		**Not disabled**		**Disabled**		**P-value**
			**(mRS 0–2; N = 1286)**		**(mRS 3–5; N = 679)**		
Educational attainment							
Low/less educated (≤ high school)	1131	57.6	677	52.6	454	66.9	<.001
More (> high school)	834	42.4	609	47.4	225	33.1	
Working status							<.001
Working pre-stroke	767	39.0	593	46.1	174	25.6	
Retired	865	44.0	528	41.1	337	49.6	
Disabled and not working	164	8.4	82	6.4	82	12.1	
Unemployed/homemaker	169	8.6	83	6.5	86	12.7	
Perceived adequacy of household income: Inadequate	533	27.1	290	22.5	243	35.8	<.001
Adequate income	1432	72.9	996	77.5	436	64.2	
Age, Mean – STD	65.5	13.7	64.2	13.3	68.1	14.0	<.001
Median - IQR	66.0	56.0-76.0	65.0	55.0-74.0	69.0	58.0-80.0	
Gender							
Female	869	44.2	513	39.9	356	52.4	<.001
Male	1095	55.7	772	60.0	323	47.6	
Race/Ethnicity,							
White	1626	82.8	1081	84.1	545	80.3	0.005
Black or African American	222	10.6	125	9.1	97	13.4	
Asian	21	1.1	11	0.9	10	1.5	
Am. Indian/Alaska Native	9	0.5	8	0.6	1	0.2	
Native Hawaiian/Pacific Is.	4	0.2	1	0.1	3	0.4	
Hispanic	54	2.8	31	2.4	23	3.4	
Other, unable to determine	37	1.9	31	2.4	6	0.9	
Previous stroke or transient ischemic attack, Yes	427	23.7	237	20.4	190	30.0	<.001
Diabetes mellitus, Yes	559	31.1	334	28.7	225	35.4	0.003
Hypertension, Yes	1408	78.2	895	76.9	513	80.7	0.064
Smoker, Yes	474	26.3	303	26.0	171	26.9	0.694
Symptom onset to tPA time in minutes, Mean – STD	134.7	33.3	132.7	35.4	137.7	29.8	0.412
(Number treated with tPA)	(167)		(101)		(66)		
Stroke severity (NIHSS Score)*, Mean – STD	5.0	5.1	4.0	4.3)	6.9	(5.9)	<.001
Ambulatory status at discharge, **Independent	1209	61.5	936	72.8	273	40.2	<.001
With person assist	548	27.9	258	20.1	290	42.7	
Unable to ambulate	108	5.5	25	1.9	83	12.2	
Discharge destination							<.001
Home	1172	59.6	929	72.2	243	35.8	
Rehabilitation	611	31.1	283	22.0	328	48.3	
Skilled nursing facility	130	6.6	47	3.7	83	12.2	
Left AMA	2	0.1	2	0.2	0	0.0	
Transfer to acute care	38	1.9	20	1.6	18	2.7	
Hospice	1	0.05	0	0.00	1	0.2	

*29.7% missing data for NIHSS.

**5.1% missing data for ambulatory status at discharge or not documented.

*mRS* Modified Rankin Scale Score, *NIHSS* NIH Stroke Scale, *IV tPA* intravenous tissue plasminogen activator.

A third of the sample had post-stroke disability (34.6%). Compared with patients with a mRS of 0–2, those with significant disability at 3-months were older, female, had a history of diabetes mellitus or prior stroke or transient ischemic attack, had more severe strokes, required assistance or were unable to ambulate at hospital discharge, and were discharged to a post-acute service setting (inpatient rehabilitation or skilled nursing facility).

### Education

More than half (57.6%) of stroke patients in this sample completed high school or less education. Among those with disability at 3 months, 66.9% had less education and 33.1% had more than a high school education (p < 0.001; Table 1). In unadjusted analyses (Table 2), stroke patients with lower education were more likely to be disabled at 3-months compared to those with more than a high school education (odds ratio (OR) = 1.82, 95% confidence interval (CI) 1.50-2.20). Although the odds ratio decreased, lower education remained independently associated with higher odds of disability after adjusting for age, gender and race (OR = 1.70, 95% CI 1.40-2.07), and after adjusting for demographic and clinical factors (OR = 1.44, 95% CI 1.12-1.85).

**Table 2:** Unadjusted and adjusted odds of disability (mRS 3–5) 3-months post-stroke for each indicator of socioeconomic status among ischemic stroke patients in AVAIL (OR, 95% CI)

Socioeconomic factors*	**Unadjusted OR (95% CI)**	**Adjusted for demographics**^**† **^**OR (95% CI)**	**Adjusted for demographics + Clinical**^**‡ **^**OR (95% CI)**
	**(N = 1965)**	**(N = 1956)**	**(N = 1380)**
Educational attainment (ref. = > high school)			
Less educated (≤ high school)	1.82 (1.50-2.20)	1.70 (1.40-2.07)	1.44 (1.12-1.85)
Working status (ref. = working pre-stroke)			
Retired	2.18 (1.75-2.70)	1.82 (1.40-2.36)	1.87 (1.34-2.60)
Disabled and not working	3.41 (2.40-4.84)	3.20 (2.25-4.56)	2.46 (1.58-3.83)
Unemployed/homemaker	3.53 (2.50-4.99)	2.93 (2.04-4.21)	3.19 (2.02-5.02)
Perceived adequacy of household income (ref. = had adequate income)			
Had inadequate income	1.91 (1.56-2.35)		
Had inadequate income at age ≤ 55		3.28 (2.14-5.02)	2.81 (1.64-4.83)
Had inadequate income age 56-70		2.39 (1.72-3.32)	2.60 (1.71-3.95)
Had inadequate income at age > 70		1.14 (0.78-1.65)	1.03 (0.65-1.62)

*Each socioeconomic status factor was modeled independent of the others.

^†^Demographic factors: age, gender and race.

^‡^Clinical factors: history of stroke or transient ischemic attack, co-morbid diabetes mellitus, co-morbid hypertension, current smoker, treatment with intravenous tissue plasminogen activator, stroke severity (NIH Stroke Scale score), and discharge ambulatory status.

*mRS* Modified Rankin Scale Score.

### Working status

Almost half of the patients (44.0%) were retired prior to their stroke, 8.4% disabled and not working, 8.6% were unemployed or homemakers, and 39.0% were working. After adjusting for demographic and clinical factors (Table 2), those who were retired (OR = 1.87, 95% CI 1.34-2.60), disabled and not working (OR = 2.46, 95% CI 1.58-3.83), or unemployed/homemakers pre-stroke (OR = 3.19, 95% CI 2.02-5.02), were more likely to be disabled than those who were working pre-stroke.

### Household income

Despite almost two thirds not working pre-stroke, only 27.1% of patients indicated inadequate household income pre-stroke. Disability at 3-months was reported by 30.4% (N = 436) of those with adequate income but 45.6% (N = 243) with inadequate income. In unadjusted analyses (Table 2), patients who reported inadequate household income pre-stroke were more likely to be disabled than those with adequate income (OR = 1.91, 95% CI 1.56-2.35). After adjusting for demographic and clinical factors and the interaction between income and age, the odds of disability increased among those with inadequate income and 55 years of age or younger (OR = 2.81, 95% CI 1.64-4.83); inadequate income and 56–70 years of age (OR = 2.60, 95% CI 1.71-3.95); and was no longer significant for patients older than 70 years of age.

### Combined impact of socioeconomic status and disability

In a combined model with all three measures of SES (Table 3), work status and income for patients 70 years and younger but not education were independently associated with the outcome after adjusting for patient demographics and clinical characteristics. Patients with inadequate income and 70 years or younger (≤55 or 56–70 years), or who were retired, disabled and not working, or unemployed/homemakers pre-stroke were more likely to be disabled at 3-months. VIF for each variable in the final model was < 2 indicating correlation between variables is low. Compared to a model with only the three measures of SES (c-index of 0.656), the final model’s discriminatory ability was good with a c-index of 0.747.

**Table 3:** Final model for odds of disability (mRS 3–5) 3-months post-stroke with 3 indicators of socioeconomic status (N = 1380)

	**Chi-Square**	**AOR (95% CI)**	**P-value**
Educational attainment (ref. = > high school):			
Less educated (≤ high school)	2.75	1.25 (0.96-1.62)	0.097
Working status (ref. = working pre-stroke):			
Retired	13.24	1.87 (1.34-2.62)	<.001
Disabled and not working	7.51	1.90 (1.20-3.00)	0.006
Unemployed/homemaker	18.74	2.78 (1.75-4.42)	<.001
Perceived adequacy of household income (ref. = had adequate income):			
Had inadequate income at age ≤ 55 years	10.18	2.46 (1.42-4.27)	0.001
Had inadequate income at age 56–70 years	14.75	2.34 (1.52-3.61)	<.001
Had inadequate income at age > 70 years	0.04	1.05 (0.66-1.67)	0.833
Gender: Female (vs. Male)	11.73	1.56 (1.21-2.01)	<.001
Race: White (vs. Other)	3.37	0.74 (0.53-1.02)	0.066
Medical history:			
Previous stroke or transient ischemic attack	7.81	1.52 (1.13-2.05)	0.005
Diabetes mellitus	0.38	1.09 (0.82-1.45)	0.540
Hypertension	1.05	1.17 (0.87-1.57)	0.306
Smoker	1.06	1.18 (0.86-1.61)	0.304
Stroke severity: NIHSS Score (per 1 unit increase)	50.38	1.10 (1.07-1.13)	<.001
Treated with IV tPA: Yes (vs. not treated)	2.42	0.71 (0.46-1.09)	0.120
No due to contraindication (vs. not treated)	10.54	0.63 (0.48-0.83)	0.001
Ambulatory status at discharge: Independent (vs. with assistance or unable)	26.15	0.36 (0.24-0.53)	<.001

*mRS* Modified Rankin Scale Score, *NIHSS* NIH Stroke Scale, *IV tPA* intravenous tissue plasminogen activator.

### Death and dependence

Thirty-six percent of patients (N = 727) were dead or dependent 3-months after hospital discharge (Additional file 1: Table S1). Findings for the examination of each SES factor adjusted for demographics and clinical characteristics (Additional file 2: Table S2) were similar to the examination of disability at 3-months with increased odds of death or dependence for patients with less education, who were retired, disabled and not working, or unemployed or a homemaker pre-stroke, or reported inadequate income and were 55 years and younger or 56–70 years of age. In the combined model with all three measures of SES (Additional file 3: Table S3), patients with inadequate income and were 70 years or younger (≤55 or 56–70 years), or who were retired, disabled and not working, or unemployed/homemakers pre-stroke were more likely to be disabled at 3-months.

## Discussion

This is the first study of the association of three indicators of SES and disability in a large U.S. cohort of stroke survivors. In this study, lower educational attainment, unemployment and inadequate income were each independently associated with a worse functional outcome. Examination of multiple indicators of SES collectively found stroke survivors with lower SES as measured by working status and perceived income were more likely to be disabled at 3-months after their stroke. Identifying patients who are of lower SES has important implications for providing patient-centered interventions in the post-acute period of recovery.

To our knowledge, our study is the first to examine pre-stroke working status as associated with post-stroke recovery. Adults in this cohort working prior to their stroke had the greatest odds of a more positive outcome even after adjusting for stroke severity. Other indicators of SES have been examined for their relationship with function and disability, but other U.S. cohorts and studies of European registries provide little opportunity to directly compare with our findings [13-17]. For example, in Switzerland patients with private insurance had less severe strokes and better outcomes (mRS ≤ 2) at 7 days and 3 months than those with basic insurance in a universal health care system [13]. Comparable research of U.S. stroke patients might be worthwhile to explore in the future in states that have more recently provided universal access to health care. We were unable to examine health insurance in this study as it wasn’t a required variable in the clinical registry.

Education and income are more commonly studied but with conflicting results when considering SES more broadly. In a large study in Finland (N = 6,903), fewer patients with high income needed assistance with activities of daily living 28 days after their stroke [15]. In a smaller sample in the Netherlands (N = 465), there was a non-significant trend toward less disability (Barthel Index and mRS) at 6 months and 5 years post-stroke among those with higher education [16]. A significant relationship between higher education and a favorable functional outcome (Barthel Index) was found for 1688 patients in one hospital participating in the Berlin Stroke Registry [17]. Among stroke patients in six European stroke rehabilitation units, educational level was a determinant of recovery during inpatient rehabilitation. However, income and not education predicted recovery after discharge from inpatient rehabilitation [14]. We describe each individual study because comparison across all patient samples studied to-date is limited as a result of different measures of SES (insurance, income, and education) for stroke patients recruited from different settings (acute and rehabilitation care), and analysis of different outcome measures (Barthel Index and mRS) measured at different time points post-stroke.

Stroke patients with low SES and at higher risk for disability may be among the most vulnerable patients as they are likely unable to compensate for the loss in function and have a steeper trajectory of functional decline. Future research on recovery trajectories is needed to identify factors that might affect the rate and extent of recovery for patients of higher and lower SES. In doing so, research could identify protective factors that enable some patients to stay healthy despite the increased risk associated with low SES. This association of SES and functional outcomes may have important implications for hospital and rehabilitation quality improvement, for discharge planning to support stroke survivors as they transition home, and for public health services in place to improve community re-integration and independent living.

Although there is a trend toward improved quality of acute stroke care in the U.S. [18-21], only in European samples have differences in processes and quality of care been identified as predictors of functional outcomes and that they differ for patients with low compared to high SES [13,15]. Quality, continuity, and coordination of care for all stroke patients is important for preventing adverse events and readmissions and improving functional outcomes and independence; however, our study suggests research is needed to determine whether targeted interventions for stroke patients with low SES could improve adherence to secondary prevention and rehabilitation strategies, adjustment with the transition to home, and re-integration with community and vocational pursuits. There may be an opportunity to further engage community services and primary care providers to explore the differences in lifestyle behaviors related to functional outcomes, for example physical activity post-stroke, among stroke survivors with lower versus higher SES.

Several limitations of our study warrant discussion. Although pre-morbid disability is known to be associated with functional outcomes, a specific measure was not a part of this clinical registry and longitudinal study. Available to us was a measure of independence with ambulation prior to hospital admission; however, these data were missing for 36% of the sample and among those with data, 98% were independent. Alternatively, we delineated those patients who were not working prior to their stroke due to disability and as expected, found these patients to be at increased odds of disability and death and dependence 3-months post-stroke. Stroke severity as measured by the NIHSS was not available for the entire sample as participation in GWTG is voluntary and not all data are required to be completed; however, the availability of stroke severity was not associated with the outcome, thus reducing the likelihood of selection bias in this study. Additionally, participating hospitals may not be representative of the overall U.S. hospital population since early GWTG-Stroke participants (2006) likely had a stronger interest in stroke and quality improvement. No external audit of case ascertainment is in place and variation in patient selection at the local hospital-level could have occurred. Previous GWTG studies have shown that patient admissions in GWTG appear to be representative of the overall U.S. stroke population in terms of age, demographics and medical comorbidities [22]. By design, this study did not consider factors such as resource utilization or complications that occurred between hospital discharge and the outcome assessment at 3 months. Although these factors can influence functional recovery, the intent was to understand the importance of pre-stroke measures of SES independent of the critical decisions made during hospital discharge planning and post-acute care. Finally, our primary analysis excluded patients who died prior to the 3-month interview recognizing that they clearly exhibited a path of functional decline but stroke outcome studies have shown that the factors associated with mortality are different than those associated with functional status, the focus of our study. We included an exploration of the outcome death and dependence to allow for comparison with other studies that chose to include those who died in their patient sample. The findings for the two outcomes, disability among survivors and death or dependence, were similar.

## Conclusion

In our national cohort of stroke survivors, lower pre-stroke SES was associated with a less favorable outcome, disability and death or dependence 3-months after a stroke. Stroke survivors who were working pre-stroke, had 13 or more years of education, and adequate pre-stroke income were less likely to be disabled at 3-months compared to those who were retired, disabled and not working, unemployed or homemakers, had a high school or less education and inadequate financial resources. These findings establish an important foundation for future health services, population and patient-level research. Implications from our study suggest a need for patient-centered interventions that promote the greatest opportunities for maximizing functional independence and recovery post-stroke.

## Abbreviations

AVAIL: Adherence eValuation after ischemic stroke longitudinal; SES: Socioeconomic status; U.S.: United States; mRS: Modified rankin scale; GWTG-Stroke: Get with the guidelines – stroke; NIHSS: National Institutes of Health Stroke Scale; OR: Odds ratio; CI: Confidence interval.

## Competing interests

The authors declare that they have no competing interests.

## Authors’ contributions

JPB contributed to the design of this study and the interpretation of the data. XZ contributed to the design of this study, analysis of the data, and interpretation of the data. CB contributed to the conception, design and acquisition of the data, design of this study and interpretation of the data. LZ contributed to the design of this study. WP contributed to the design of this study, analysis of the data, and interpretation of the data. LSW contributed to the interpretation of the data. EDP contributed to the conception, design and acquisition of the data, design of this study, and interpretation of the data. All authors contributed to the development of the draft, revised, and final manuscript. All authors read and approved the final approval.

## Pre-publication history

The pre-publication history for this paper can be accessed here:

http://www.biomedcentral.com/1471-2458/14/281/prepub

## Supplementary Material

Additional file 1: Table S1 Patient Characteristics Associated with 3-month Death and Dependence (N, %). Click here for file

Additional file 2: Table S2 Unadjusted and Adjusted Odds of Death and Dependence (mRS 3-6) 3-months Post-stroke for Each Indicator of Socioeconomic Status Among Ischemic Stroke Patients in AVAIL (OR, 95% CI). Click here for file

Additional file 3: Table S3 Final Model for Odds of Death and Dependence (mRS 3-6) 3-months Post-stroke with 3 Indicators of Socioeconomic Status (N = 1421). Click here for file
