# Supplementary material for: The association between socioeconomic status and disability after stroke: Findings from the Adherence eValuation After Ischemic stroke Longitudinal (AVAIL) registry
Source: BMC Public Health. 2014 Mar 26;14:281. doi: 10.1186/1471-2458-14-281 (PMC3987648; doi:10.1186/1471-2458-14-281)
Supplement: Additional file 1: Table S1 — Patient Characteristics Associated with 3-month Death and Dependence (N, %). [file 1471-2458-14-281-S1.doc]

**Additional file 1: Table S1: Patient Characteristics Associated with 3-month Death and Dependence (N, %)**

| **Variable** | **Total N=2022** | | **Not Disabled**  **(mRS 0-2; N=1295)** | | **Death and Dependence**  **(mRS 3-6; N=727)** | | **P-value** |
| --- | --- | --- | --- | --- | --- | --- | --- |
| Educational Attainment, Low /  Less Educated (≤ high school) | 1169 | 57.8 | 684 | 52.8 | 485 | 66.7 | <.001 |
| More (> high school) | 853 | 42.2 | 611 | 47.2 | 242 | 33.3 |  |
| Working Pre-stroke | 777 | 38.4 | 596 | 46.0 | 181 | 24.9 | <.001 |
| Perceived Adequacy of Household Income: Inadequate | 553 | 27.4 | 294 | 22.7 | 259 | 35.6 | <.001 |
| Adequate Income | 1469 | 72.7 | 1001 | 77.3 | 468 | 64.4 |  |
| Age, Mean (STD) | 65.8 | (13.7) | 64.2 | (13.3) | 68.6 | (13.9) | <.001 |
| Median (IQR) | 66.0 | (57.0-77.0) | 65.0 | (55.0-74.0) | 69.0 | (59.0-80.0) |  |
| Gender, Female | 896 | 44.3 | 517 | 39.9 | 379 | 52.1 | <.001 |
| Male | 1125 | 55.6 | 777 | 60.0 | 348 | 47.9 |  |
| Race/Ethnicity, |  |  |  |  |  |  |  |
| White | 1677 | 82.9 | 1088 | 84.0 | 589 | 81.0 | 0.008 |
| Black or African American | 211 | 10.4 | 121 | 9.3 | 90 | 12.4 |  |
| Hispanic | 55 | 2.7 | 31 | 2.4 | 24 | 3.3 |  |
| Other (Asian, Am. Indian / Alaska Native, Native Hawaiian/Pacific Is., Unable to Determine) | 71 | 3.5 | 51 | 3.9 | 20 | 2.8 |  |
| Previous Stroke or Transient Ischemic Attack, Yes | 449 | 24.2 | 241 | 20.6 | 208 | 30.6 | <.001 |
| Diabetes Mellitus, Yes | 575 | 31.1 | 335 | 28.6 | 240 | 35.4 | 0.002 |
| Hypertension, Yes | 1450 | 78.3 | 903 | 77.0 | 547 | 80.6 | 0.072 |
| Smoker, Yes | 481 | 26.0 | 304 | 25.9 | 177 | 26.1 | 0.943 |
| Symptom Onset to tPA  Time in minutes, Mean (STD) | 134.9 | (33.3) | 132.7 | (35.4) | 138.2 | (29.7) | 0.343 |
| (Number Treated with tPA) | (169) |  | (101) |  | (68) |  |  |
| Stroke Severity (NIHSS Score)*, Mean (STD) | 5.0 | (5.1) | 4.0 | (4.3) | 6.8 | (5.9) | <.001 |
| Ambulatory Status at Discharge,** Independent | 1225 | 60.6 | 937 | 72.4 | 288 | 39.6 | <.001 |
| With person assist | 571 | 28.2 | 262 | 20.2 | 309 | 42.5 |  |
| Unable to ambulate | 122 | 6.0 | 28 | 2.2 | 94 | 12.9 |  |
| Discharge Destination |  |  |  |  |  |  | <.001 |
| Home | 1191 | 58.9 | 934 | 72.1 | 257 | 35.4 |  |
| Rehabilitation | 633 | 31.3 | 285 | 22.0 | 348 | 47.9 |  |
| Skilled Nursing Facility | 141 | 7.0 | 48 | 3.7 | 93 | 12.8 |  |
| Left AMA | 2 | 0.1 | 2 | 0.2 | 0 | 0.0 |  |
| Transfer to Acute Care | 41 | 2.0 | 21 | 1.6 | 20 | 2.8 |  |
| Hospice | 2 | 0.10 | 0 | 0.00 | 2 | 0.3 |  |

*29.7% missing data for NIHSS

**5.1% missing data for ambulatory status at discharge or not documented

mRS = Modified Rankin Scale Score; NIHSS = NIH Stroke Scale; IV tPA = intravenous tissue plasminogen activator.
