# Supplementary material for: The association between socioeconomic status and disability after stroke: Findings from the Adherence eValuation After Ischemic stroke Longitudinal (AVAIL) registry
Source: BMC Public Health. 2014 Mar 26;14:281. doi: 10.1186/1471-2458-14-281 (PMC3987648; doi:10.1186/1471-2458-14-281)
Supplement: Additional file 2: Table S2 — Unadjusted and Adjusted Odds of Death and Dependence (mRS 3-6) 3-months Post-stroke for Each Indicator of Socioeconomic Status Among Ischemic Stroke Patients in AVAIL (OR, 95% CI). [file 1471-2458-14-281-S2.doc]

**Additional file 2: Table S2**. Unadjusted and Adjusted Odds of Death and Dependence (mRS 3-6) 3-months Post-stroke for Each Indicator of Socioeconomic Status Among Ischemic Stroke Patients in AVAIL (OR, 95% CI)

| Socioeconomic Factors* | Unadjusted OR (95% CI)  (N=2022) | Adjusted for Demographics^†^  (OR (95%CI)  (N=2013) | Adjusted for Demographics +Clinical^‡^  OR (95% CI)  (N=1421) | |
| --- | --- | --- | --- | --- |
| Educational Attainment (ref. = > high school) |  |  |  |  |
| Less Educated (≤ high school) | 1.81 (1.50-2.19) | 1.70 (1.41-2.06) | 1.46 (1.15-1.87) |  |
| Working Status (ref. = working pre-stroke) |  |  |  |  |
| Retired | 2.28 (1.85-2.82) | 1.85 (1.43-2.39) | 1.97 (1.42-2.72) |  |
| Disabled and Not Working | 3.50 (2.48-4.93) | 3.32 (2.35-4.69) | 2.55 (1.65-3.95) |  |
| Unemployed / homemaker | 3.46 (2.46-4.86) | 2.84 (1.99-4.05) | 3.16 (2.02-4.95) |  |
| Perceived Adequacy of Household Income (ref. = had adequate income) |  |  |  |  |
| Had Inadequate Income | 1.91 (1.56-2.33) |  |  |  |
| Had Inadequate Income at Age<55 |  | 3.22 (2.18-4.90) | 2.79 (1.64-4.75) |  |
| Had Inadequate Income Age 56-70 |  | 2.36 (1.71-3.27) | 2.70 (1.79-4.09) |  |
| Had Inadequate Income at Age>70 |  | 1.24 (0.87-1.77) | 1.16 (0.75-1.79) |  |

* Each socioeconomic status factor was modeled independent of the others

† Demographic factors: age, gender and race

‡ Clinical factors: history of stroke or transient ischemic attack, co-morbid diabetes mellitus, co-morbid hypertension, current smoker, treatment with intravenous tissue plasminogen activator, stroke severity (NIH Stroke Scale score), and discharge ambulatory status

mRS = Modified Rankin Scale Score
