# Supplementary material for: The association between socioeconomic status and disability after stroke: Findings from the Adherence eValuation After Ischemic stroke Longitudinal (AVAIL) registry
Source: BMC Public Health. 2014 Mar 26;14:281. doi: 10.1186/1471-2458-14-281 (PMC3987648; doi:10.1186/1471-2458-14-281)
Supplement: Additional file 3: Table S3 — Final Model for Odds of Death and Dependence (mRS 3-6) 3-months Post-stroke with 3 Indicators of Socioeconomic Status (N = 1421). [file 1471-2458-14-281-S3.doc]

**Additional file 3: Table S3.** Final Model for Odds of Death and Dependence (mRS 3-6) 3-months Post-stroke with 3 Indicators of Socioeconomic Status (N=1421)

|  | Chi-Square | AOR (95% CI) | P-value |
| --- | --- | --- | --- |
| Educational Attainment (ref. = > high school):  Less Educated (≤ high school) | 3.13 | 1.26 (0.98-1.62) | 0.077 |
| Working Status (ref.= working pre-stroke):  Retired | 16.38 | 1.98 (1.42-2.77) | <.001 |
| Disabled and not working | 8.40 | 1.95 (1.24-3.07) | 0.004 |
| Unemployed / homemaker | 18.73 | 2.75 (1.74-4.36) | <.001 |
| Perceived Adequacy of Household Income (ref. = had adequate income):  Had Inadequate Income at Age < 55 years  Had Inadequate Income at Age 56-70 years  Had Inadequate Income at Age > 70 years | 10.24  16.81  0.53 | 2.43 (1.41-4.19)  2.45 (1.60-3.76)  1.18 (0.76-1.84) | 0.001  <.001  0.467 |
| Gender: Female (vs. Male) | 10.26 | 1.50 (1.17-1.93) | <.001 |
| Race: White (vs. Other) | 2.58 | 0.77 (0.56-1.06) | 0.108 |
| Medical History: |  |  |  |
| Previous Stroke or Transient Ischemic Attack | 7.80 | 1.51 (1.13-2.01) | 0.005 |
| Diabetes Mellitus | 0.40 | 1.09 (0.83-1.44) | 0.526 |
| Hypertension | 0.42 | 1.10 (0.82-1.47) | 0.519 |
| Smoker | 0.81 | 1.15 (0.85-1.57) | 0.369 |
| Stroke Severity: NIHSS Score (per 1 unit increase) | 47.79 | 1.10 (1.07-1.13) | <.001 |
| Treated with IV tPA: Yes (vs. Not treated)  No due to Contraindication (vs. Not treated) | 3.14  10.42 | 0.68 (0.44-1.04)  0.64 (0.49-0.84) | 0.077  0.001 |
| Ambulatory Status at Discharge: Independent (vs. with Assistance or Unable) | 32.33 | 0.33 (0.28-0.49) | <.001 |

mRS = Modified Rankin Scale Score; NIHSS = NIH Stroke Scale; IV tPA = intravenous tissue plasminogen activator
